# Population structure and genetic bottleneck in sweet cherry estimated with SSRs and the gametophytic self-incompatibility locus

**DOI:** 10.1186/1471-2156-11-77

**Published:** 2010-08-20

**Authors:** Stéphanie Mariette, Muriel Tavaud, Uraiwan Arunyawat, Gaëlle Capdeville, Muriel Millan, Franck Salin

**Affiliations:** 1 INRA, Unité de Recherche sur les Espèces Fruitières, Domaine de la Grande Ferrade, 71 avenue Edouard Bourlaux, BP 81, 33883 Villenave d'Ornon Cedex, France; 2 INRA, UMR GDPP, Domaine de la Grande Ferrade, 71 avenue Edouard Bourlaux, BP 81, 33883 Villenave d'Ornon Cedex, France; 3 INRA, UMR DIAPC, Domaine de Melgueil, 34130 Mauguio, France; 4 Centre technique interprofessionel des fruits et légumes, DSTFL-équipe Fruits, Centre Ctifl de Balandran, BP32, 30127 Bellegarde, France; 5 INRA, UMR BIOGECO 1202, Domaine de l'Hermitage, BP45, 33611 Gazinet-Cestas Cedex, France; 6 Department of Genetics, Faculty of Science, Kasetsart University, Bangkok 10900, Thailand; 7 INRA, UMR GDPP, Domaine de la Grande Ferrade, 71 avenue Edouard Bourlaux, BP 81, 33883 Villenave d'Ornon Cedex, France

## Abstract

**Background:**

Domestication and breeding involve the selection of particular phenotypes, limiting the genomic diversity of the population and creating a bottleneck. These effects can be precisely estimated when the location of domestication is established. Few analyses have focused on understanding the genetic consequences of domestication and breeding in fruit trees. In this study, we aimed to analyse genetic structure and changes in the diversity in sweet cherry *Prunus avium *L.

**Results:**

Three subgroups were detected in sweet cherry, with one group of landraces genetically very close to the analysed wild cherry population. A limited number of SSR markers displayed deviations from the frequencies expected under neutrality. After the removal of these markers from the analysis, a very limited bottleneck was detected between wild cherries and sweet cherry landraces, with a much more pronounced bottleneck between sweet cherry landraces and modern sweet cherry varieties. The loss of diversity between wild cherries and sweet cherry landraces at the *S*-locus was more significant than that for microsatellites. Particularly high levels of differentiation were observed for some *S*-alleles.

**Conclusions:**

Several domestication events may have happened in sweet cherry or/and intense gene flow from local wild cherry was probably maintained along the evolutionary history of the species. A marked bottleneck due to breeding was detected, with all markers, in the modern sweet cherry gene pool. The microsatellites did not detect the bottleneck due to domestication in the analysed sample. The vegetative propagation specific to some fruit trees may account for the differences in diversity observed at the *S*-locus. Our study provides insights into domestication events of cherry, however, requires confirmation on a larger sampling scheme for both sweet cherry landraces and wild cherry.

## Background

Most of the edible species were domesticated from wild species, generally several thousand years ago. The first cultivated populations directly sampled from wild populations were then improved, through breeding, to obtain landraces, which were further selected to produce modern varieties. The evolutionary history of a cultivated species results from a complex interaction between genetic and demographic factors. This history can be precisely rebuilt when the location of early domestication is known. A single origin is generally hypothesized, and was demonstrated for example in maize [1]. However, in other crop species, such as barley, multiple domestications explain genetic data better than a single domestication event [1].

Domestication and breeding have two major impacts on the diversity of plant genomes. Firstly, traits convenient for human use, such as development of organs used by man, or adaptation to new environments, have been selected, resulting in selection signatures at specific loci. For example, an analysis of single nucleotide polymorphisms (SNP) in 774 maize genes showing 2 to 4% of these genes had been subject to artificial selection [2]. The second major impact is a bottleneck affecting the entire genome, due to the demographic sampling of individuals during domestication and breeding. This process decreases diversity. However, the degree of diversity loss differs considerably between crop plants: 34% based on SNP diversity in soybean and 38% in maize, but as much as 70 to 90% in wheat (69% in bread wheat and 84% in durum wheat) and 80 to 90% in rice, depending on the sample studied and the molecular markers used [3-6].

Genetic mutation may also modify the diversity of cultivated plants. A general loss of diversity at all genetic loci is expected when a wild plant is domesticated, but some loci may display new variation due to mutations occurring after domestication. The observed loss of diversity may also differ between loci in the genome and, consequently, between molecular markers, as a function of mutation rates. Such differences have been observed for microsatellite markers, in particular. Dinucleotide microsatellites underestimate the bottleneck in maize, due to a high rate of mutation, regenerating diversity at these loci after domestication [7]. In this case, comparing with teosinte only a moderate loss of diversity is observed in maize (19%). Similarly, in sorghum, a post-domestication increase in diversity has been observed for some genes [8].

Moreover, domestication may lead to reproductive isolation between the domesticated form and its wild relative (e.g. mating system modification, geographic isolation). However, gene flow between cultivated and wild forms after domestication, even at very low levels, may have a marked impact on the dynamics of cultivated plant diversity [9,10].

Despite the worldwide cultivation of fruit trees, few studies have analysed the genetic domestication and breeding history of these species. The most obvious change between wild forest species and fruit trees is the change of reproduction system. Fruit tree crops are generally propagated vegetatively, rather than by seed [11]. Many fruit trees were cultivated in classical times, (e.g. olive, grapevine, fig, date palm, pomegranate, sycamore fig), but others were domesticated much later (e.g. apple, pear, plum, cherry), probably because grafting is necessary for the cultivation of these species [12]. As grafting has been used to propagate selected individuals, it is thought that cultivated clones persisted for many years, and few genetic changes due to selection would therefore be expected [12]. However, breeding programmes based on selection were investigated for several fruit species in the 20^th ^Century, with the aim of improving fruit quality and disease resistance. The impact of human selection over the last few decades may therefore be much greater. The use of grafting may have led to the propagation of a limited number of highly interesting genotypes, provoking a marked bottleneck in these species. Furthermore, a limited number of varieties in modern selection programmes may also result in a stronger bottleneck due to breeding.

Sweet cherry is the domesticated form of wild cherry (*Prunus avium *L.). The fruits of the domesticated and wild form are very similar but differ by the size of the fruit and of the stone [12]. As wild cherry is distributed throughout Europe, human populations probably picked wild cherries in forests long before they began cultivating sweet cherry fruit trees. Sweet cherry cultivation was introduced in Europe by the Romans [12]. Pliny the Elder (23-79 AD) wrote in his *Natural history *that the Roman general Lucullus brought cherries back to Italy when he returned from the Pontus region in Turkey. Based on morphological and genetic studies, there is evidence that sweet cherry was present in Europe in Roman and early medieval times [13-15]. Consequently, the proof of a single domestication event was not established for this species. Multiple domestications events and introgression from the wild form after domestication cannot be excluded.

Moderate genetic differentiation between populations was observed in wild cherry, based on several isozymes and microsatellites studies [16-19]. Recent studies have also provided insight into the reproduction system of this species. Clonal reproduction by root suckering has been observed in many populations, accounting for the heterozygote excess observed within populations [16,20-24]. Sexual reproduction is controlled by a Gametophytic Self-Incompatibility (GSI) system, which is now well described in this species [19,21,25-27].

The genetic diversity of sweet cherry varieties has also been analysed with both dominant and codominant markers [28-31]. Comparisons of wild and cultivated cherry pools on the basis of chloroplast DNA led to the identification of 16 haplotypes in wild cherries and three haplotypes in sweet cherries, suggesting a marked bottleneck in the species [32]. Even there are several studies on cherry but still little is known about cherry domestication and improvement.

Therefore in this study, we aimed to analyse the genetic structure of sweet cherry and assess the impacts of selection and bottlenecks in sweet cherry. Based on *Prunus avium *L. genetic material conserved in French repositories, we addressed this issue using two types of marker: microsatellites or SSRs and the *S*-locus. We chose this last locus because the number of *S*-alleles is directly linked to effective population size [33-35]. Besides, it can be hypothesized that domestication and breeding may have been modified the frequency of allele, according to varieties that were propagated and crosses that were performed among genotypes. We specifically addressed the following questions:

1/ what is the genetic structure of sweet cherry landraces and modern varieties conserved in French repositories?

2/ is there any genetic contribution of French wild cherry to analysed sweet cherries?

3/ what is the genetic variation among the identified genetic pools, as estimated with SSRs and the *S*-locus?

## Results

### Cherry genetic structure

**Figure 1:**
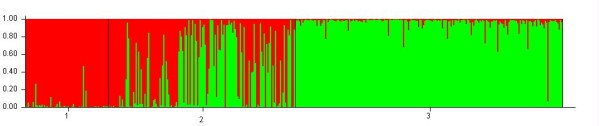
**Structure bar plot results obtained on the whole set of data at K = 2**. 1 is population identification for modern varieties, 2 is population identification for landraces and 3 is population identification for wild cherries.

When Structure was performed on the whole data set, a maximum value of the rate of change in the log probability of data was revealed at K = 2, using Evanno's method [36]. For K = 2, wild cherry was composed of one unambiguous population, with some significant admixture from the second population for some individuals (e.g. one wild cherry individual being clearly from the second population). Modern sweet cherries were the second population with some admixture from the first population. Obviously, landraces were a mixture of individuals from both populations (Figure 1).

**Figure 2:**
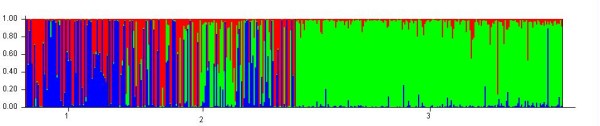
**Structure bar plot results obtained on the whole set of data at K = 3**. 1 is population identification for modern varieties, 2 is population identification for landraces and 3 is population identification for wild cherries.

For K = 3, the results were congruent, suggesting however a more complex structure of sweet cherries with three possible groups: one group linked with the wild cherry pool (green on Figure 2) and two other groups (red and blue). For this analysis, landraces were composed of individuals from the three groups (red blue and green), and modern varieties only from two groups (red and blue). The group of wild cherry (green) was admixed by the two groups of sweet cherries, and reversely, the two groups of modern sweet cherries were admixed by wild cherry (see Additional file 1, Table S1). Pairwise *F*_ST _values provide additional information on the relationships among the identified sub-populations. Comparing wild cherry and landraces, the lowest *F*_ST _value (0.022) was logically observed between wild cherry and landrace 3 group, suggesting less differentiation between them, whereas landrace 1 group was the most differentiated group from wild cherry (*F*_ST _~ 0.10, this value being significantly different from the *F*_ST _calculated between wild cherry and landrace 3 group), and the differentiation between landrace 2 group and wild cherry was intermediate. Moreover, the high *F*_ST _values between the wild cherry group and the two groups of modern varieties also confirmed that modern varieties are not genetically based on this origin (Table 1).

**Table 1:** Pairwise *F*_ST _and confidence interval calculated on subpopulations defined by Structure analysis

	Landrace 1	Landrace 2	Landrace 3	Modern 1	Modern 2	Wild cherry
Landrace 1		0.03-0.06	0.05-0.10	0.03-0.09	0.05-0.12	0.07-0.12

Landrace 2	0.05		0.02-0.04	0.06-0.13	0.03-0.08	0.03-0.06

Landrace 3	0.08	0.03		0.09-0.16	0.05-0.10	0.01-0.03

Modern 1	0.06	0.09	0.12		0.04-0.10	0.10-0.18

Modern 2	0.08	0.05	0.07	0.07		0.06-0.11

Wild cherry	0.10	0.05	0.02	0.14	0.09	

*F*_ST _(below diagonal) and confidence intervals (above diagonal) were estimated using the Genetix 4.05 software [48].

**Figure 3:**
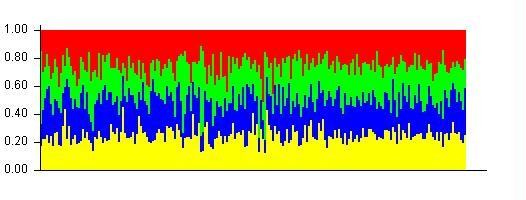
Structure bar plot results obtained on the wild cherry at K = 4

When Structure was then run on wild cherries only, the more probable number of populations was 4. However, considering the results, each individual was an admixture of all populations with equal contribution of each population (Figure 3). This was also true for K = 2 and K = 3. Therefore, the wild cherry pool was considered as unstructured population.

When Structure was run on sweet cherries and landraces separately, the more probable number of populations was 2, with groups in correspondence with the results obtained on the pooled data set for K = 3 (Figure not shown). Lastly, when Structure was performed on modern varieties, the most likely number of populations was 2, with groups in correspondence with the results obtained on the pooled data set for K = 3 (Figure not shown).

Interestingly, the first subgroup of individuals in modern varieties included all cultivars coming from the Summerland (Canada) breeding program. Moreover, many self-compatible cultivars were in this group, having then Stella, the first self-compatible cultivar, as a parent or grand-parent. Also, when parents of this first group were known, we found that Van variety was parent of about 10% of trees.

The second modern varieties subgroup was formed of cultivars with different origins (France, USA but none of them coming from Canada) with Burlat variety being the parent for 15% of varieties.

Based on Structure results, wild cherry was constituted of only one population, three groups in landraces and two groups in modern varieties were distinguished. The group for each studied sweet cherry individual is provided in Additional file 2, Table S2. The individuals were attributed to each group based on the results within each group, and confirmed by comparing the results with the analysis on the complete data set. Individuals were assigned to a group when the results obtained on the complete data set and on each cherry group were congruent. Note that some landraces were assigned as Lmixed group because the results from both analyses were not congruent. These strongly admixed landraces (Lmixed group) were removed from further analyses.

### Estimation of genetic diversity in the cherry genetic pools

The average number of alleles, means of observed and expected heterozygosities as well as inbreeding coefficient are reported in Table 2.

**Table 2:** Average estimations of genetic diversity in wild cherry and sweet cherry genetic pools

		A	**H**_**O**_	**H**_**E**_	***F***_**IS**_
Wild cherry	Mean (all SSRs)	9	0.65	0.68	0.04

	Mean (SSRs developed in *P. avium*)	8.79	0.64	0.66	0.03

	Mean (SSRs developed in other species)	9.25	0.65	0.69	0.06

	Mean (dinucleotide SSRs)	8.86	0.62	0.65	0.05

	Mean (complex repeat motif SSRs)	8.10	0.67	0.70	0.04

	*S*-locus	19	1.00	0.93	-0.08

Sweet cherry	Mean (all SSRs)	7.85	0.63	0.63	-0.00

	Mean (SSRs developed in *P. avium*)	7.64	0.63	0.62	-0.02

	Mean (SSRs developed in other species)	8.08	0.64	0.64	0.02

	Mean (dinucleotide SSRs)	7.64	0.58	0.59	0.03

	Mean (complex repeat motif SSRs)	7	0.68	0.65	-0.04

	*S*-locus	15	1.00	0.86	-0.16

Landraces	Mean (all SSRs)	7.62	0.66	0.64	-0.02

	Mean (SSRs developed in *P. avium*)	7.36	0.65	0.63	-0.03

	Mean (SSRs developed in other species)	7.92	0.66	0.65	0.01

	Mean (dinucleotide SSRs)	7.57	0.59	0.60	0.04

	Mean (complex repeat motif SSRs)	6.70	0.73	0.67	-0.09

	*S*-locus	15	1	0.86	-0.16

Modern varieties	Mean (all SSRs)	4.12	0.59	0.56	-0.05

	Mean (SSRs developed in *P. avium*)	4.29	0.57	0.54	-0.08

	Mean (SSRs developed in other species)	3.92	0.61	0.59	-0.04

	Mean (dinucleotide SSRs)	4.07	0.56	0.52	-0.08

	Mean (complex repeat motif SSRs)	3.80	0.58	0.57	-0.03

	*S*-locus	9	1	0.84	-0.19

A is the allelic richness (number of alleles), H_O _is the observed heterozygosity, H_E _is the unbiased expected heterozygosity and *F*_IS _is the inbreeding coefficient, as calculated in the Genetix 4.05.2 software [48].

The number of alleles was slightly lower for SSRs developed in *Prunus avium *than for SSRs developed in other species, (7.6 *vs *8.1 in wild cherry, 8.8 *vs *9.3 in sweet cherry, 7.4 *vs *7.9 in landraces), except in modern varieties (4.3 *vs *3.9). The number of alleles was also a bit lower in complex SSRs than in dinucleotide SSRs (7 *vs *7.6 in wild cherry, 8 *vs *8.9 in sweet cherry, 6.7 *vs *7.6 in landraces and 3.8 *vs *4.1 in modern varieties).

An excess of heterozygosity was observed at SSRs in landraces and modern varieties, and it was significant in modern varieties. In wild cherry, the *F*_IS _value was low but significant. As expected, the excess of heterozygosity was observed in each group at the *S*-locus.

### Deviation of markers from neutral expectations

To obtain a neutral subsample of markers, we conducted the Fdist2 analysis on three comparisons of populations based on Structure results. We compared (1) modern varieties and landraces from the first group identified with Structure, (2) modern varieties and landraces from the second group identified with Structure, and (3) the wild cherry population and landraces that grouped together with wild cherry in the Structure analysis.

**Figure 4:**
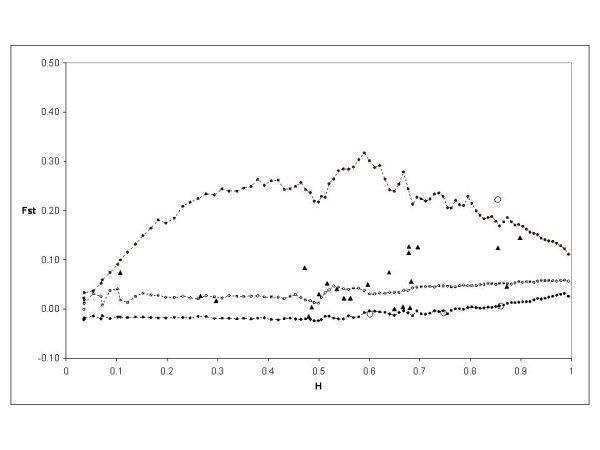
**Distribution of *F*_ST _values as a function of expected heterozygosity (H) using the *F*_ST _between modern 1 and landrace 1 groups (0.059099)**. The envelope of values corresponding to neutral expectations with the infinite allele model was constructed as described by [53]. Dotted lines with plain circles represent the 0.5(1 - 0.95) and 0.5(1 + 0.95) quantiles. Dotted lines and circles represent the median of values. Triangles represent the observations not significant at 5%. Circles represent the observations significant at 5%.

The *F*_ST _calculated by Fdist2 between Modern 1 and Landrace 1 groups was 0.059222. Based on the first analysis, four outlier loci were detected at the 95% level: PS12A02, EMPAS11, EMPAS14 and the *S*-locus. These outliers were removed for a second analysis. No outlier was detected and the *F*_ST _value was 0.059099. Then this *F*_ST _value was used to perform again the analysis, and the same four outliers were detected from this final run (Figure 4).

**Figure 5:**
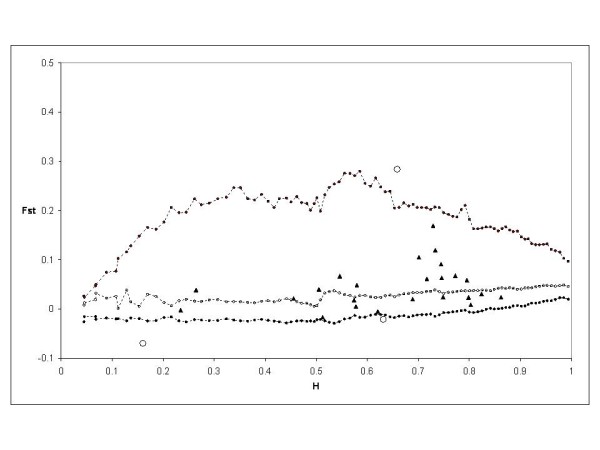
**Distribution of *F*_ST _values as a function of expected heterozygosity (H) using the *F*_ST _between modern 2 and landrace 2 groups (0.048377)**. The envelope of values corresponding to neutral expectations with the infinite allele model was constructed as described by [53]. Dotted lines with plain circles represent the 0.5(1 - 0.95) and 0.5(1 + 0.95) quantiles. Dotted lines and circles represent the median of values. Triangles represent the observations not significant at 5%. Circles represent the observations significant at 5%.

A similar analysis was done on Modern 2 and Landrace 2 groups. The *F*_ST _was 0.053816 and three outliers were detected at the 95% level: PCHGMS1, UDP98409 and EMPA026. After removing these three loci, the *F*_ST _value was 0.048377 and no outlier was detected. However, the same three outliers were detected in the last analysis using the *F*_ST _value of 0.048377 (Figure 5).

**Figure 6:**
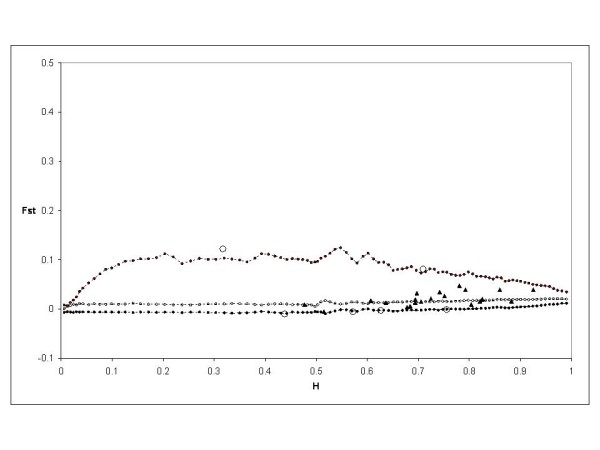
**Distribution of *F*_ST _values as a function of expected heterozygosity (H) using the *F*_ST _between wild cherries and modern sweet cherry varieties (0.021650)**. The envelope of values corresponding to neutral expectations with the infinite allele model was constructed as described by [53]. Dotted lines with plain circles represent the 0.5(1 - 0.95) and 0.5(1 + 0.95) quantiles. Dotted lines and circles represent the median of values. Triangles represent the observations not significant at 5%. Circles represent the observations significant at 5%.

Finally, using the same procedure, the *F*_ST _between Landrace 3 and wild cherry groups was 0.022269. Six outliers were detected at the 95% level: EMPA002, PCHGMS1, EMPAS11, UDP96001, EMPA004 and UDP98409. The *F*_ST _after removing them was 0.021650 and the same six outliers were detected using this last value of *F*_ST _(Figure 6).

### Estimation of the relative loss of diversity due to domestication and breeding

#### Allelic richness

A larger relative loss of diversity was observed for the *S*-locus (24%) than for microsatellites (3%), when wild cherry was compared with landraces. A similar result was obtained when comparing wild cherry with the third group of landraces (Table 3).

**Table 3:** Relative loss of diversity due to domestication

	**A**_**R**_		**H**_**O**_		**H**_**E**_	
**Number of alleles**	***S*-locus**	**SSRs**	***S*-locus**	**SSRs**	***S*-locus**	**SSRs**

Relative loss of diversity (Wild cherry/All landraces)	0.24	0.03	0	-0.04	0.07	0.05

Relative loss of diversity (Wild cherry/Landrace 3)	0.22	-0.04	0	-0.04	0.08	0

The loss of diversity was estimated with the allelic richness (A_R_), the observed heterozygosity (H_O_) and the expected heterozygosity (H_E_) in wild cherry, landraces and the third group of landraces (Landrace 3). It was estimated using all SSRs markers for the comparison between the wild cherry and all landraces, and using only markers under the assumption of neutrality for the comparison between wild cherry and the third group of landraces.

Breeding had a stronger impact on diversity than domestication (Table 4). Comparisons of landraces with modern varieties revealed a loss of diversity of about 30% for the *S*-locus, whatever the comparison (all landraces compared with all modern varieties, or modern 1 with landrace 1, and modern 2 with landrace 2). For microsatellites, the loss of diversity was about 40% comparing between all landraces and all modern varieties, and only 13% and 12% for landrace1/modern1 and landrace2/modern2 comparisons, respectively.

**Table 4:** Relative loss of diversity due to breeding

	**A**_**R**_		**H**_**O**_		**H**_**E**_	
**Number of alleles**	***S*-locus**	**SSRs**	***S*-locus**	**SSRs**	***S*-locus**	**SSRs**

Relative loss of diversity (Landraces/Modern varieties)	0.29	0.38	0	0.09	0.02	0.14

Relative loss of diversity (Landrace 1/Modern 1)	0.34	0.13	0	0.20	0.06	0.11

Relative loss of diversity (Landrace 2/Modern 2)	0.26	0.12	0	0.05	0.05	0.10

The loss of diversity was estimated with the allelic richness (A_R_), the observed heterozygosity (H_O_) and the expected heterozygosity (H_E_) in landraces, in modern varieties and in the sub-populations detected by Stucture. The relative loss of diversity was estimated using all SSRs markers for the comparison between the landraces and modern varieties, and using only markers under the assumption of neutrality for the comparisons between Landrace 1 and Modern 1, and between Landrace 2 and Modern 2.

#### Observed heterozygosity

Unlike the number of alleles, the observed level of heterozygosity was somewhat affected by domestication and breeding. The *S*-locus is always heterozygous, certainly there was no loss of observed heterozygosity, whatever the comparison (Table 3 and Table 4). For microsatellites, no loss of observed heterozygosity was observed when comparing wild cherry and landraces. A moderate decrease in microsatellite observed heterozygosity was observed when modern varieties were compared to landraces (9%), this loss being higher when comparing landrace 1 and modern 1 (20%) and slightly smaller when comparing landrace 2 and modern 2 (5%).

#### Expected heterozygosity

Unlike the decrease in the number of alleles, expected heterozygosity at the *S*-locus decreased smoothly (between 2% and 8%). The loss of expected heterozygosity was also moderate for microsatellites for comparisons of wild cherry and landraces (5% and even no loss when comparing wild cherry and Landrace 3). This loss was larger for comparisons of landraces and modern varieties (between 10 and 14%).

### Diversity and differentiation at the gametophytic self-incompatibility locus

We identified all the *S*-alleles described in wild and cultivated *Prunus avium *L., with the exception of S27 to S32, which were originally described in non-native material in England and probably originate from Middle Eastern countries [37]. In the sweet cherry landraces pool, S17, S20, S21 and S22 alleles were identified twice, three times, twice and once, respectively.

Table 5 shows the level of genetic differentiation at the *S*-locus. The *F*_ST _between wild cherry and sweet cherry landraces was 0.05 (the *F*_ST _was 0.04 when considering wild cherry and landrace 3 groups). The differentiation between sweet cherry landraces and modern sweet cherry varieties was small (*F*_ST _= 0.02), likewise between landrace 1 and modern 1 (*F*_ST _= 0.01) and between landrace 2 and modern 2 (*F*_ST _= 0.03). In addition, *F*_ST _varied considerably between alleles at this locus. High *F*_ST _values (>0.10) were observed for alleles S1, S3 and S4 and moderate values (between 0.05 and 0.10) were observed for alleles S2 and S9.

**Table 5:** *F*_ST _values at each allele of the *S*-locus

Allele	***F***_**ST **_**(W-L)**	***F***_**ST **_**(W-L3)**	***F***_**ST **_**(L-M)**	***F***_**ST **_**(L1-M1)**	***F***_**ST **_**(L2-M2)**
S1	0.01	0.06	0.06	0.01	0.12

S2	0.06	0.06	0.08	-0.01	0.06

S3	0.11	0.14	0.00	-0.01	-0.01

S4	0.11	0.09	-0.01	-0.01	0.03

S4'	NA	NA	0.09	0.05	0.01

S5	0.02	0.01	0.02	0.14	0.04

S6	0.02	0.05	0.01	-0.01	0.01

S7	0.02	-0.01	0.02	0.01	-0.01

S9	0.09	-0.01	0.00	0.01	-0.01

S10	0.04	0.03	NA	NA	NA

S12	0.01	0.02	0.03	0.01	-0.01

S13	0.00	0.00	0.00	0.01	-0.01

S14	0.04	0.00	0.00	-0.02	-0.01

S16	0.01	0.00	NA	NA	NA

S17	0.04	0.02	0.00	0.01	NA

S18	0.02	0.01	NA	NA	NA

S19	0.02	0.01	NA	NA	NA

S20	0.00	0.00	0.00	NA	-0.01

S21	0.03	0.00	0.00	NA	NA

S22	0.03	0.01	0.00	NA	NA

All	0.05	0.04	0.02	0.01	0.03

The level of genetic differentiation for each allele at the *S*-locus was estimated with the Fstat 2.9.3.2 software [52]. W is for wild cherry, L is for landraces, M for modern varieties; L1, L2 and L3 are respectively for the group 1, group 2 and group 3 of landraces as defined by the Structure software; M1 and M2 are respectively for the group 1 and group 2 of modern varieties as defined by the Structure software.

## Discussion

### One or several events of domestication of sweet cherries?

According to Pliny the Elder, cherry did not exist in Italy prior to its introduction by Lucullus from Turkey (*Natural history*, 15^th ^book). This unique reference about cherry cultivation during the Roman times implies a single introduction of cherries into western European countries, and consequently a domestication in the Middle East. When considering the analysis of the whole set of data with K = 2, our analysis suggested that cherries were composed of two main groups, with admixture of the two groups for many individuals. One of the groups was genetically identical to the wild cherry we analysed and the other was independent. When considering the analysis with three populations, up to three groups of cherries were detected, but one of the groups was still genetically identical to the wild cherry group. Two scenarios can be deduced from our results. Either a single event of domestication and then intense gene flow from wild cherry occurred, forming differentiated groups of landraces, or secondary domestications occurred based on western wild cherry material. When looking carefully at Figure 2, we can hypothesize that both gene flow and secondary domestications may have happened. Indeed, some landraces are a clear admixture between the wild and sweet cherries groups. Moreover, some other landraces are clearly attributed to the group of wild cherries. Instead of a single domestication event, the domestication of sweet cherry would then be a complex process, that may include several origins and may result from clonal propagation of desirable genotypes and sexual reproduction with local wild cherry. This would be congruent with the hypothesis developed by other authors to explain the evolutionary history of clonally propagated domesticated plants [38].

Concerning the differentiation of material, groups 1 and 2 of sweet cherries were genetically different from the wild cherry we analysed, whereas the third group was genetically close to the wild cherry group. Nevertheless, the differentiation between wild cherry and the first group of landraces was much higher than the differentiation with the second group of landraces (analysis with K = 3, differentiation in Table 1). We interpreted our results cautiously since we knew that our sampling was limited to the material conserved in French repositories. However, the differentiation of wild cherry in the western part of Europe is low [18]. Thus, under the assumption that the wild cherry we sampled is a good genetic representation of western Europe, the origin of the first group of cherries should be searched in Caucasia or Middle East countries. The origin of the second group of cherries may be geographically closer. Also the genetic differentiation that was observed between sweet cherries and wild cherry may result from the drift on allelic frequencies due to domestication and breeding. Our study may not offer the final conclusion because of the sampling limitation.

### A significant loss of alleles during domestication and breeding

Domestication and breeding generally cause a loss of diversity due to bottleneck and genetic drift. We performed two types of bottleneck analyses. First, we assumed that a comparison of wild cherry and sweet cherry landraces could be used to estimate the domestication bottleneck, whereas a comparison between sweet cherry landraces and modern sweet cherry varieties would estimate the breeding bottleneck. However, these comparisons can be biased by selection and by the presence of structure in the genetic groups. Within population structure can also influence the detection of selection [39]. Therefore, we also compared diversity within the different groups identified by the Structure analyses after removing outlier loci (wild cherry/landrace 3 to assess the domestication bottleneck, modern 1/landrace 1 and modern 2/landrace 2 to assess the breeding bottleneck). Results were congruent among the two types of analyses since the bottleneck associated with cherry domestication was much weaker than that associated with modern breeding, for all SSR markers and all diversity estimators (allelic richness, observed heterozygosity, expected heterozygosity). The magnitude of the breeding bottleneck was obviously different among the two types of analyses (pooled *versus *within group). This difference is due to the diversity present in the third group of landraces that increased the diversity in the whole group of landraces when comparing all modern varieties *versus *all landraces.

Diversity after a bottleneck depends on the ratio of wild population size to cultivated population size and the duration of the bottleneck [4,5]. In the case of cherry, as for other fruit trees, the bottleneck is probably recent because domestication occurred relatively late, so the duration of the bottleneck is likely to have been short [12]. If domestication took place in several places, there may also have been a large number of founders, accounting for the small loss of diversity. By contrast, modern breeding has had a significant impact on the level of diversity, with as many as 40% of the alleles present in wild cherry lost in modern cherry varieties, for all microsatellites tested. This finding suggests that only small numbers of individuals are used as parents in cherry breeding programs. For example, in North American breeding programs, only five founding clones are frequently used, resulting in high levels of inbreeding [40]. The use of a small number of clones in modern cherry breeding may account for the higher level of allele differentiation observed for some alleles of the *S*-locus than for this locus itself.

Nevertheless, our sampling was geographically limited, with the wild cherry sample taken from French populations only. We may therefore have underestimated the bottleneck due to domestication. Based on cpDNA markers, only three haplotypes were identified in sweet cherry cultivars and up to 16 haplotypes in wild cherry populations, suggesting a much more severe bottleneck [32]. However, their sweet cherry sample included mostly modern breeding varieties, accounting for this discrepancy. Further studies should include samples covering a larger geographic distribution of wild cherries, because genetic diversity in Caucasia (Georgia) may be greater than that in Europe [18] [Frédérique Santi, personal communication]. Local landraces from many countries should also be included, as far as possible. Additionally, it would be useful to identify the potential ancestors of sweet cherries among wild cherry populations, to obtain more insight into the genetic bottleneck.

### Specific impact of domestication and breeding on genetic diversity at the *S*-locus

In the present study, we found 19, 15 and 9 *S*-alleles in wild cherry, landraces and modern varieties, respectively. Consequently, based on our analysis, the domestication bottleneck was about 20%, the breeding bottleneck was about 30% and the total bottleneck was approximately 50%.

Thirteen alleles are usually described in cultivated sweet cherry, plus the S4' allele generated by mutation in the sweet cherry pool [41]. These alleles, except S4', also present in wild cherry together with two additional series: S17 to S22 in Belgian wild cherries, and S27 to S32 in non-native wild cherries from Middle Eastern origin [25,37]. Then up to now, 25 alleles were described in wild cherry. Considering between 13 alleles in sweet cherry and 25 alleles in wild cherry, the total bottleneck at the *S*-locus is about 50%. Interestingly, this approximation is very similar to our estimate based on a limited sampling.

In this study, we identified several previously undescribed alleles (S17, S20, S21 and S22) in the sweet cherry landraces pool. Similarly, S19 and S22, which have previously been identified mainly in wild cherry, were only found to be present at very low frequency in a German sweet cherry collection [42]. Thus, as for SSRs, the breeding bottleneck significantly reduced the diversity at the *S*-locus now used in breeding program.

Though the exact bottleneck due to domestication only is difficult to estimate, the loss of diversity at the *S*-locus with respect to wild cherry was greater than that at microsatellites, with a loss of 22-24% for the *S*-locus in sweet cherry landraces. This observation may be explained by the change of reproduction regime occurring during domestication, with the introduction of grafting -- a vegetative propagation technique -- and the limited use of seed production for cherry crops and for other fruit trees. Two studies have suggested that the number of alleles at a self-incompatibility locus should decrease with increasing clonality within populations [43,44]. Indeed, balancing selection on the *S*-locus, which promotes high allelic diversity, occurs only during sexual reproduction events. Then allelic diversity at the *S*-locus is expected low in highly clonal populations. This effect is reinforced further in conditions of strong drift [44]. By contrast, no loss of alleles with increasing clonality would be expected at neutral loci, since polymorphism is protected within individuals due to fixed heterozygosity [45]. The use of grafting may therefore help to account for the continuous loss of diversity observed at the *S*-locus, illustrating the modification of evolutionary dynamics of sexual reproduction expected in clonally propagated crop species [38].

## Conclusions

Several domestication events may have happened in sweet cherry or/and intense gene flow from local wild cherry was probably maintained along the evolutionary history of the species. A marked bottleneck due to breeding was detected with all markers in the modern sweet cherry gene pool. The bottleneck due to domestication was not picked up with microsatellites, in the sample analysed here. The vegetative propagation specific to some fruit trees may explain the loss of diversity observed at the *S*-locus during domestication.

This study provides insights into domestication events and evolutionary history of cherry, however, requires confirmation on a larger sampling scheme for both sweet cherry landraces and wild cherry.

## Methods

### Plant materials

We assessed sweet cherry diversity based on 207 varieties sampled from the INRA Bordeaux Prunus Genetic Resources Centre, the INRA Bordeaux sweet cherry breeding collection and the CTIFL collection. This sample consisted of 141 landraces and 66 modern varieties. Many landraces were of unknown origin, but the sample included varieties from very probable origin in France, Belgium, the Czech Republic, Germany, Hungary, Spain, Italy, Iran, Romania and Turkey. The origin of modern varieties was more evident since they were obtained from various breeding programs (Australia, Canada, the Czech Republic, France, Italy, and USA). Detailed information on varieties was provided in Additional file 2, Table S2. The 211 studied wild cherry individuals were sampled from the INRA Orléans collection. Trees were originally collected in France, with sampling from most regions.

### Choice of markers and molecular genotyping

Individuals were genotyped for 26 SSRs and the gametophytic self-incompatibility locus. For SSRs, markers were chosen on the basis of the ease of amplification in cherry, their location on *Prunus *maps and the type of polymorphism of each marker (dinucleotides or other repeats). The list of SSR markers is given in Table 6.

**Table 6:** Information concerning the markers used

Marker name	Linkage group	Repeat motif	Reference
UDP96005	1	(AC)_16_TG(CT)2CA(CT)_11_	[54]

EMPA002	1	(AG)_13_	[55]

EMPA003	1	(AC)_8_	[55]

EMPA005	1	(CT)_3_CAT(CT)_12_T(AC)_23_	[55]

PCHGMS1	2	(AC)_12_(AT)_6_	[56]

PCEGA34	2	NA	[57]

UDP98411	2	(TC)_16_	[58]

BPPCT034	2	(GA)_19_	[46]

EMPA017	2	(AG)_19_	[55]

EMPaS02	3	(TTG)_7_ctgc(TG)_10_(AG)_8_	[59]

EMPaS12	3	(TG)_10_a/GA)_10_aa(GA)_13_	[59]

PS12A02	4	NA	[60]

BPPCT040	4	(GA)_14_	[46]

EMPaS06	4	(CT)_12_	[59]

EMPaS10	4	(GA)_28_	[59]

EMPaS11	5	(TC)_25_	[59]

EMPaS14	5	(TC)_10_ccat(TC)_5_ccat(TC)_8_	[59]

UDP96001	6	(CA)_17_	[54]

UDP98021	6	(GA)_22_(CA)_11_	[58]

UDP98412	6	(AG)_28_	[58]

EMPA004	6	(GA)_4_AA(GA)_4_AA(GA)_15_	[55]

EMPaS01	6	(GA)_9_(GA)_11_	[59]

UCDCH14	7	(CT)_18_	[30]

UDP98409	8	(AG)_19_	[54]

EMPA018	8	(GA)_18_	[55]

EMPA026	8	complex (CT)	[61]

Linkage group position is given based on information published for a cherry map (inter-specific cross *Prunus avium *'Napoleon' × *P. nipponica*), except for BPCCT034 for which the position is given based on a work published on another cherry map [61,62].

The repeat motif of each marker is given based on information provided, or not, by the original reference that published primers for the marker.

For six markers (UDP96001, UDP96005, UDP98409, PCHGMS1, PCEGA34 and PS12A02), we used data obtained by simplex amplification and silver staining analysis [18,46]. For the other 20 markers, the protocol was as follows. DNA was extracted from leaves using the DNeasy^® ^96 plant kit (QIAGEN, Germany). Multiplex PCR was carried out with the Type It Microsatellite PCR Kit^® ^(QIAGEN, Germany). PCR was carried out in a volume of 20 μl, containing 4 ng genomic DNA, 1× multiplex PCR master mix and between 0.12 and 0.48 μM of each primer. Tests were first carried out to optimise the amount of primer used for each marker. Four multiplex PCRs were used to amplify all 20 markers. PCR conditions were as recommended by the kit manufacturers. The PCR products were diluted (1/400), subjected to agarose gel electrophoresis and analysed with an ABI 3730 sequencer, according to the manufacturer's instructions. Allele sizes were determined with Genemapper software (Applied Biosystems, USA).

We analysed variability at the gametophytic self-incompatibility locus, using fluorescently labelled primers to amplify both the SFB intron and the first intron of the S-RNase gene[26,27]. For each sweet cherry landrace, the genotypes of which had not previously been determined for these two loci, data were acquired from two independent clones (two trees of the same landrace were sampled and two DNA extractions, two PCR amplifications and two sequencer runs were then carried out). The runs were independently read by two people, to confirm the results obtained.

### Structure analysis of the genetic pool

In order to understand the distribution of genetic diversity in our sample, we used a model-based clustering approach as implemented in Structure software version 2.3.1 to infer population structure of wild and sweet cherries [47]. For each analysis, Structure was run with different values of the number of clusters (K) varying from 1 to 8 under the admixture model with no prior population information. To verify the consistency of the results, we performed 10 independent runs per K value with 50,000 burn-in period and 50,000 MCMC replications. The run with the maximum likelihood was used to assign the most probable number of subpopulations, which was determined using an ad hoc statistic based on the rate of change in the log probability of data between successive K values [36].

The Structure analysis was performed on the whole dataset to test whether wild and sweet cherries pools can be identified. It was run afterwards on four separate groups: 1/ wild cherries, 2/ sweet cherries, and, within sweet cherries, 3/ landraces, 4/ modern varieties.

To assess the level of differentiation among sub-groups identified by Structure, pairwise *F*_ST _and confidence intervals were calculated using Genetix 4.05 software [48].

### Analysis of the genetic variation among identified genetic clusters

After characterising the genetic structure of cherries, our second aim was to assess the bottleneck possibly due to breeding and domestication. However, strictly speaking, the domestication bottleneck estimation should be done using the wild cherry sub-population from which domestication was performed. Similarly, the breeding bottleneck can be estimated either considering the whole set of landraces and modern varieties or comparing between landraces and modern varieties from the same ancestral group.

Besides, selection is an evolutionary force that can affect the estimation of bottleneck, and a natural set of markers should be obtained before analysing bottleneck due to successive samplings. Nonetheless, within population structure can modify the detection of selected locus [39].

Consequently, after the basic analysis of diversity in each group (wild cherry, sweet cherry, landraces, modern varieties), we performed two types of comparisons. First we compared diversity (1) between wild cherry and all landraces and (2) between landraces and modern varieties, without testing for selection because of sub-structure in landraces and modern varieties. Second, after testing markers for selection, we compared one of the group of landraces with wild cherry since these two groups were genetically connected (see the Results section), modern varieties and landraces from the same first group (see the Results section), and finally modern varieties and landraces from the same second group (see the Results section).

#### Basic diversity analysis

The number of alleles (A), the allelic richness (A_R_), the observed heterozygosity (H_O_), the unbiased heterozygosity (H_E_) and the inbreeding coefficient *F*_IS _were assessed for each locus on wild cherry, sweet cherries, modern varieties and landraces, but also on each group as defined using Structure (two groups of modern varieties and three groups of landraces). See definitions of estimators in [49-51]. Mean values and standard deviations were calculated for each population.

Since markers were developed on different species, and this may influence marker polymorphims, we also compared the mean values between the set of markers developed in sweet cherries and the set of markers developed in other species. Finally, we also calculated the mean values of dinucleotide markers and the mean values of complex repeat motif.

Most estimators were calculated using Genetix 4.05 software, except for the allelic richness that was estimated using Fstat 2.9.3.2 software [48,52].

#### Constituting sub-samples of neutral markers

Selection at or near a marker may affect the diversity and differentiation of that marker, accounting for some of the observed variation between groups. Since we are interested in characterizing the variation of diversity mainly due to domestication or breeding bottleneck, we thus tested for selection to define a sub-sample of neutral markers on which we made the genetic diversity analysis. For this purpose we used the method developed by [53]. This method involves the detection of unusually high or low levels of *F*_ST_, by plotting *F*_ST _against heterozygosity on the set of markers.

As we compared diversity between wild cherry, landraces and modern varieties, Fdist2 was conducted on three population pairs: 1/ wild cherry/landraces that may originate from France (see the Results section), and two comparisons of landraces and modern varieties with the same origin (see the Results section). For each comparison, we ran 50,000 simulations using the infinite allele model for markers. A first analysis revealed a first set of outliers. They were removed and a new *F*_ST _was calculated, which was used to make a new analysis, revealing a possible second set of outliers. The analysis was iterated until no further locus fell outside of the expected distribution. The last value of *F*_ST _was used as the neutral value to detect outliers on the whole set of data.

#### Diversity comparisons

The relative loss of diversity was estimated based on neutral markers as defined before. The relative loss of diversity was estimated with three diversity estimators: the allelic richness, the observed heterozygosity and the expected heterozygosity as described by [7]. For each estimator, the relative loss of diversity was estimated by calculating 1-(DIV_1_/DIV_2_), where DIV_1 _is the estimator of diversity in the supposed derivating genetic pool and DIV_2 _is the estimator of diversity in the supposed originating genetic pool. The relative loss of diversity was estimated between wild cherry and landraces and between landraces and modern varieties. The relative loss of diversity was also estimated between the wild cherry and the landraces that may originate from France (see the Results section), between landraces and modern varieties with the same origin (see the Results section).

### Allelic composition and differentiation at the gametophytic self-incompatibility locus

The frequency of each *S*-allele was calculated in each group (wild cherry, landraces and modern varieties and also in each subgroup indentified by Structure). Moreover, the level of genetic differentiation for each allele at the *S*-locus was estimated using Fstat 2.9.3.2 software [52].

## Authors' contributions

SM conceived of the study, participated in molecular genetic studies and in statistical analyses and drafted the manuscript. MT participated in the design of the study, participated in molecular genetic studies and helped to draft the manuscript. UA participated in statistical analyses and helped to draft the manuscript. GC carried out the molecular genetic studies. MM participated in the design of the study and participated in *S*-locus characterization. FS participated in the design of the molecular genetic studies. All authors read and approved the final manuscript.

## Supplementary Material

Additional file 1 **Table S1. Information on the level of admixture in groups defined with the Structure software**. Proportion of membership of each pre-defined population in each of the three clusters (results from one run of Structure on the complete dataset). Colors (green, red and blue) refer to Figure 2. Click here for file

Additional file 2 **Table S2. Information on studied sweet cherry varieties**. The code indicates the reference of the variety in the national register of introduction for cherries. The name is the one with which the variety was introduced in the INRA (or CTIFL) collection. The exact origin is given when known by the authors. A large group of studied landraces are included in the "French cherries national collection" but this means that varieties were usually cultivated in France, this does not mean that they were domesticated in France, or created in France. The partition between landrace and modern was done based on the available information on varieties (varieties known before the 20^th ^breeding programs were put in the landrace group and varieties developed after, and especially recent hybrids, were put in the modern group). The group was assigned based on Structure analysis within each cherry group, and confirmed by comparing the results with the analysis on the complete data set. Individuals were assigned to a group when the results obtained on the complete data set and on each cherry group were congruent. M1 and M2 are the two groups identified for modern varieties, L1, L2 and L3 are the three groups identified for landraces. Note that some landraces were assigned as Lmixed group because the results from both analyses were not congruent. Click here for file
