# Supplementary material for: Population structure and genetic bottleneck in sweet cherry estimated with SSRs and the gametophytic self-incompatibility locus
Source: BMC Genet. 2010 Aug 20;11:77. doi: 10.1186/1471-2156-11-77 (PMC2933703; doi:10.1186/1471-2156-11-77)
Supplement: Additional file 1 — Table S1. Information on the level of admixture in groups defined with the Structure software. Proportion of membership of each pre-defined population in each of the three clusters (results from one run of Structure on the complete dataset). Colors (green, red and blue) refer to Figure 2. [file 1471-2156-11-77-S1.DOC]

**Additional file 1. Table S1 – Proportion of membership of each pre-defined population in each of the three clusters (results from one run of Structure on the complete dataset). Colors (green, red and blue) refer to Figure 2.**

| Population | 1 (green) | 2 (red) | 3 (blue) |
| --- | --- | --- | --- |
| Modern varieties | 0.013 | 0.412 | 0.576 |
| Landraces | 0.266 | 0.384 | 0.35 |
| Wild cherry | 0.925 | 0.049 | 0.026 |
