# Supplementary material for: Population structure and genetic bottleneck in sweet cherry estimated with SSRs and the gametophytic self-incompatibility locus
Source: BMC Genet. 2010 Aug 20;11:77. doi: 10.1186/1471-2156-11-77 (PMC2933703; doi:10.1186/1471-2156-11-77)
Supplement: Additional file 2 — Table S2. Information on studied sweet cherry varieties. The code indicates the reference of the variety in the national register of introduction for cherries. The name is the one with which the variety was introduced in the INRA (or CTIFL) collection. The exact origin is given when known by the authors. A large group of studied landraces are included in the "French cherries national collection" but this means that varieties were usually cultivated in France, this does not mean that they were domesticated in France, or created in France. The partition between landrace and modern was done based on the available information on varieties (varieties known before the 20th breeding programs were put in the landrace group and varieties developed after, and especially recent hybrids, were put in the modern group). The group was assigned based on Structure analysis within each cherry group, and confirmed by comparing the results with the analysis on the complete data set. Individuals were assigned to a group when the results obtained on the complete data set and on each cherry group were congruent. M1 and M2 are the two groups identified for modern varieties, L1, L2 and L3 are the three groups identified for landraces. Note that some landraces were assigned as Lmixed group because the results from both analyses were not congruent. [file 1471-2156-11-77-S2.DOC]

**Additional file 2. Table S2 - Information on studied sweet cherry varieties.**

| Code | Name | Origin | Landrace/Modern | Group |
| --- | --- | --- | --- | --- |
| 88 | Cypres | National Collection | Landrace | L3 |
| 89 | Bruelles | National Collection | Landrace | L3 |
| 102 | Bigarreau Noir | National Collection | Landrace | L1 |
| 114 | Pellisier | National Collection | Landrace | L2 |
| 162 | Noir de Chlumac | National Collection | Landrace | L1 |
| 166 | Douce du champ de l'air | National Collection | Landrace | L2 |
| 167 | Noire de Boccard | National Collection | Landrace | L2 |
| 182 | Noir de Pitfam | National Collection | Landrace | Lmixed |
| 223 | De Mezel | National Collection | Landrace | L2 |
| 226 | Grand | National Collection | Landrace | Lmixed |
| 232 | Hative de Bâle | National Collection | Landrace | Lmixed |
| 262 | Maria Gaucher | National Collection | Landrace | Lmixed |
| 305 | Etienne Thuilleaux | National Collection | Landrace | L2 |
| 370 | Burlat | National Collection | Landrace | L2 |
| 372 | Précoce d'Isigny | National Collection | Landrace | L3 |
| 566 | Abbesse d'Oignies | National Collection | Landrace | L1 |
| 795 | Camus | National Collection | Landrace | Lmixed |
| 804 | Napoléon | National Collection | Landrace | Lmixed |
| 892 | Bigarreau De Mai | National Collection | Landrace | L3 |
| 896 | Courte queue | National Collection | Landrace | L2 |
| 897 | Cristobalina | Spain | Landrace | L3 |
| 935 | Guillaume | National Collection | Landrace | Lmixed |
| 990 | Précoce Bernard | National Collection | Landrace | L2 |
| 1360 | Ferbolus | National Collection | Landrace | L1 |
| 1378 | NA | USA | Modern | M1 |
| 1429 | Marmotte | National Collection | Landrace | Lmixed |
| 1439 | Moreau | National Collection | Landrace | L2 |
| 1646 | Tardif Gauthier | National Collection | Landrace | L2 |
| 1657 | Beaulieu | National Collection | Landrace | Lmixed |
| 1678 | Merveille de Saint Genis Laval | National Collection | Landrace | L2 |
| 1775 | Noir d'Ecully | National Collection | Landrace | L2 |
| 1814 | Sandar | National Collection | Landrace | L2 |
| 1865 | D'Annonay | National Collection | Landrace | L2 |
| 1872 | Stark Gold Bigarreau d'or | National Collection | Landrace | L1 |
| 1877 | Hedelfingen | Germany | Landrace | L1 |
| 1890 | La Chalonnaise N°1 | National Collection | Landrace | L2 |
| 1891 | La Chalonnaise N°2 | National Collection | Landrace | L2 |
| 1903 | Van | Canada | Modern | M1 |
| 1927 | Stark | USA | Modern | M1 |
| 1929 | Belge | National Collection | Landrace | Lmixed |
| 1931 | Rainier | National Collection | Landrace | L1 |
| 2017 | Summit | Canada | Modern | M1 |
| 2030 | Tombret | National Collection | Landrace | Lmixed |
| 2033 | Camus de Venasque | National Collection | Landrace | L2 |
| 2155 | Garnet® Magar | USA | Modern | M1 |
| 2175 | NA | Italy | Modern | M2 |
| 2216 | Sunburst | Canada | Modern | M1 |
| 2300 | Fernola | National Collection | Landrace | Lmixed |
| 2315 | Fernier | France (INRA) | Modern | M1 |
| 2345 | Germersdorf | Hungary | Landrace | Lmixed |
| 2392 | Orléans | National Collection | Landrace | L1 |
| 2467 | Lapins | Canada | Modern | M1 |
| 2472 | Duroni 3 | Italy | Landrace | L2 |
| 2509 | Kordia | Czech Republic | Landrace | L2 |
| 2672 | Badacsony | Hungary | Landrace | L1 |
| 2678 | Noire de Meched | Iran | Landrace | Lmixed |
| 2680 | Arcina® Fercer | France (INRA) | Modern | M1 |
| 2683 | Gégé | National Collection | Landrace | Lmixed |
| 2775 | Rubin | Rumania | Landrace | L1 |
| 2799 | Adelise® Masdel | France | Modern | M2 |
| 2851 | Vanda | Czech Republic | Landrace | L1 |
| 2865 | Impériale | Italy | Landrace | L2 |
| 2868 | Regina | Germany | Landrace | L2 |
| 2872 | Bigarreau Moreau | National Collection | Landrace | L1 |
| 2890 | Poidel | France | Modern | M2 |
| 2940 | Sonata® Sumleta | Canada | Modern | M1 |
| 2948 | Cristalina® Sumnue | Canada | Modern | M1 |
| 2970 | Lempereur | France | Modern | M2 |
| 3003 | Techlovan | Czech Republic | Modern | M1 |
| 3028 | New Moon® Sumini | Canada | Modern | M1 |
| 3039 | NA | USA | Modern | M1 |
| 3094 | Primulat® Ferprime | France (INRA) | Modern | M2 |
| 3115 | Ferdiva | France (INRA) | Modern | M1 |
| 3122 | Fermina | France (INRA) | Modern | M1 |
| 3190 | Sweetearly® Panaro 1 | Italy | Modern | M2 |
| 3223 | Ferobri | France (INRA) | Modern | M2 |
| 3228 | Hybrid INRA | France (INRA) | Modern | M1 |
| 3239 | Ferdouce | France (INRA) | Modern | M1 |
| 3263 | Hybrid INRA | France (INRA) | Modern | M1 |
| 3268 | Canada | Canada | Modern | M1 |
| 3313 | Canada Giant® Sumgita | Canada | Modern | M1 |
| 3314 | Earlise® Rivedel | France | Modern | M2 |
| 3316 | Brooks | USA | Modern | M2 |
| 3382 | Fertard | France (INRA) | Modern | M1 |
| 3387 | Folfer | France (INRA) | Modern | M2 |
| 3467 | Hybrid INRA | France (INRA) | Modern | M2 |
| 3476 | Fertille | France (INRA) | Modern | M1 |
| 3498 | Coralise® Gardel | France | Modern | M2 |
| 3499 | Bigalise® Enjidel | France | Modern | M2 |
| 3501 | Sonnet | Canada | Modern | M1 |
| 3502 | Sweetheart® Sumtare | Canada | Modern | M1 |
| 3553 | 13 S 21-29 | Canada | Modern | M1 |
| 3556 | Symphonie® 13 S 25-25 | Canada | Modern | M1 |
| 3629 | Hybrid INRA | France (INRA) | Modern | M1 |
| 3654 | Hybrid INRA | France (INRA) | Modern | M1 |
| 3656 | Ferpin | France (INRA) | Modern | M2 |
| 3657 | Feria | France (INRA) | Modern | M1 |
| 3658 | Ferpact | France (INRA) | Modern | M2 |
| 3661 | Firm Red | USA | Modern | M2 |
| 3663 | Maraly® Early Red | USA | Modern | M2 |
| 3675 | Ziraat | Turkey | Landrace | Lmixed |
| 3692 | Hybrid INRA | France (INRA) | Modern | M1 |
| 3694 | Dure de Sauve | National Collection | Landrace | Lmixed |
| 3696 | Satin® Sumele | Canada | Modern | M1 |
| 3746 | Mariant® Giant Red | USA | Modern | M2 |
| 3768 | Belge | Belgium | Landrace | L1 |
| 3821 | Bellise® Bedel | France | Modern | M2 |
| 3822 | Skeena | Canada | Modern | M1 |
| 3855 | Bigarreau de Mezel | National Collection | Landrace | Lmixed |
| 3856 | St Georges | National Collection | Landrace | L3 |
| 3857 | Jaboulay | National Collection | Landrace | L2 |
| 3858 | Cœur de Pigeon | National Collection | Landrace | L3 |
| 3859 | Bigarreau d'Or | National Collection | Landrace | L3 |
| 3861 | Belle magnifique | National Collection | Landrace | L3 |
| 3862 | Guigne jaune donissen | National Collection | Landrace | L1 |
| 3863 | Noire d'Ecully | National Collection | Landrace | L2 |
| 3868 | Ferlizac | France (INRA) | Modern | M2 |
| 3915 | Black Star | Italy | Modern | M2 |
| 3963 | Hybrid Ctifl | France (CTIFL) | Modern | M1 |
| 3965 | Hybrid Ctifl | France (CTIFL) | Modern | M1 |
| 3966 | Hybrid Ctifl | France (CTIFL) | Modern | M1 |
| 3967 | Hybrid Ctifl | France (CTIFL) | Modern | M1 |
| 4000 | Tieton | USA | Modern | M2 |
| 4001 | Selah® | USA | Modern | M1 |
| 4061 | Cerise Guin Doux | National Collection | Landrace | L3 |
| 4062 | Guigne Ramon Olivia | National Collection | Landrace | L2 |
| 4063 | Cerise Guin Doux des charentes | National Collection | Landrace | L3 |
| 4064 | Cerise Petite précose | National Collection | Landrace | Lmixed |
| 4066 | Cerise Verdée | National Collection | Landrace | Lmixed |
| 4068 | Guigne Précoce du Pays du Blanc | National Collection | Landrace | Lmixed |
| 4069 | Guigne Précoce de la Marche | National Collection | Landrace | L1 |
| 4070 | Belle du Berry | National Collection | Landrace | L2 |
| 4073 | Marin | National Collection | Landrace | L3 |
| 4075 | Petite Noire | National Collection | Landrace | L3 |
| 4076 | Blanchère | National Collection | Landrace | L3 |
| 4077 | Muant | National Collection | Landrace | L2 |
| 4080 | Sainte Marie | National Collection | Landrace | L2 |
| 4081 | Blancale Précoce Boissière | National Collection | Landrace | L3 |
| 4082 | Cerise Blanche | National Collection | Landrace | L1 |
| 4083 | Le Floc | National Collection | Landrace | L3 |
| 4086 | Turque | National Collection | Landrace | L3 |
| 4087 | Olivette | National Collection | Landrace | L3 |
| 4088 | Guigne Boissière | National Collection | Landrace | L3 |
| 4089 | Bigarreau de Juin | National Collection | Landrace | L3 |
| 4092 | Gezia Beltza | National Collection | Landrace | L3 |
| 4093 | Granjaude Desvaux | National Collection | Landrace | L3 |
| 4094 | Targonnais | National Collection | Landrace | L3 |
| 4095 | Bigarreau de Fontainebleau | National Collection | Landrace | L1 |
| 4096 | Le Cadran | National Collection | Landrace | L3 |
| 4097 | Belliquette | National Collection | Landrace | L3 |
| 4098 | Abouriou Boissière | National Collection | Landrace | Lmixed |
| 4099 | La Carrée | National Collection | Landrace | L3 |
| 4100 | Bigarreau Coeur de Pigeon Tardif | National Collection | Landrace | L3 |
| 4101 | Bigarreau Marbré | National Collection | Landrace | L3 |
| 4102 | Chapata | National Collection | Landrace | L3 |
| 4103 | Coeur de Boeuf | National Collection | Landrace | L2 |
| 4104 | Blancale tardive Medge | National Collection | Landrace | L3 |
| 4105 | Caillou | National Collection | Landrace | L3 |
| 4106 | Cerise de Coeur | National Collection | Landrace | L1 |
| 4107 | Bigarreau Coeur | National Collection | Landrace | L1 |
| 4108 | Cerise blanche petite | National Collection | Landrace | L3 |
| 4109 | Noire molle tardive | National Collection | Landrace | L1 |
| 4111 | Guigne noire de Ruesnes | National Collection | Landrace | L2 |
| 4112 | Gros bigarreau rouge et jaune | National Collection | Landrace | L1 |
| 4113 | Blanc nez | National Collection | Landrace | L3 |
| 4114 | Bigarreau Coeur de Noyon | National Collection | Landrace | Lmixed |
| 4115 | Grosse blanche de Verchocq | National Collection | Landrace | L1 |
| 4116 | Cerise de Marchiennes | National Collection | Landrace | L2 |
| 4119 | Gascogne tardive de Seninghern | National Collection | Landrace | L3 |
| 4121 | Cerise d'Eperlecques croquante | National Collection | Landrace | L3 |
| 4122 | Bigarreau Hâtif productif | National Collection | Landrace | L1 |
| 4123 | Cacouanne blanche | National Collection | Landrace | L1 |
| 4124 | Bigarreau Reverchon | National Collection | Landrace | L2 |
| 4125 | Gros bigarreau Napoléon | National Collection | Landrace | Lmixed |
| 4126 | Cerise à Gustave | National Collection | Landrace | L3 |
| 4127 | Franc Tellier | National Collection | Landrace | Lmixed |
| 4128 | Petit Bigarreau | National Collection | Landrace | L3 |
| 4129 | Cerise noire à chaire ferme | National Collection | Landrace | L3 |
| 4130 | Rose à longue queue | National Collection | Landrace | L3 |
| 4131 | Cerise noire | National Collection | Landrace | Lmixed |
| 4132 | Aubain | National Collection | Landrace | L3 |
| 4133 | Gascogne double | National Collection | Landrace | L3 |
| 4134 | Bigarreau Coeur de Pigeon noire | National Collection | Landrace | L1 |
| 4135 | Saint Jean | National Collection | Landrace | L2 |
| 4136 | Michaude | National Collection | Landrace | Lmixed |
| 4137 | Versaillaise | National Collection | Landrace | L1 |
| 4138 | Boissonneï | National Collection | Landrace | Lmixed |
| 4139 | Bigarreau Court Picou | National Collection | Landrace | Lmixed |
| 4140 | Ogier | National Collection | Landrace | Lmixed |
| 4141 | Bigarreau Précoce de Soréde | National Collection | Landrace | Lmixed |
| 4142 | Gaujac | National Collection | Landrace | Lmixed |
| 4143 | Cerise des moissons | National Collection | Landrace | L2 |
| 4145 | Gobet | National Collection | Landrace | L1 |
| 4146 | Bigarreau Semi-hâtif | National Collection | Landrace | Lmixed |
| 4147 | Monjoude | National Collection | Landrace | L3 |
| 4149 | Pangaude | National Collection | Landrace | L2 |
| 4151 | Alpine de Provence | National Collection | Landrace | L3 |
| 4152 | Cordaba | National Collection | Landrace | L3 |
| 4153 | Blanche de Gaujac | National Collection | Landrace | Lmixed |
| 4154 | Bigarreau Saint Bruno | National Collection | Landrace | L2 |
| 4155 | Bigarreau Grande Queue | National Collection | Landrace | L2 |
| 4156 | Sweet Valentine | Australie | Modern | M1 |
| 2783/2954 | 13 S 42-49 | Canada | Modern | M1 |
| 3430/3554 | Staccato | Canada | Modern | M1 |
| 3431/3552 | 13 S 21-01 | Canada | Modern | M1 |
| 3565/2945 | Samba® Sumste | Canada | Modern | M1 |
| 3782/3917 | Grace Star | Italy | Modern | M2 |

**The code indicates the reference of the variety in the national register of introduction for cherries. The name is the one with which the variety was introduced in the INRA (or CTIFL) collection. The exact origin is given when known by the authors. A large group of studied landraces are included in the “French cherries national collection” but this means that varieties were usually cultivated in France, this does not mean that they were domesticated in France, or created in France. The partition between landrace and modern was done based on the available information on varieties (varieties known before the 20^th^ breeding programs were put in the landrace group and varieties developed after, and especially recent hybrids, were put in the modern group).**

**The group was assigned based on Structure analysis within each cherry group, and confirmed by comparing the results with the analysis on the complete data set. Individuals were assigned to a group when the results obtained on the complete data set and on each cherry group were congruent. M1 and M2 are the two groups identified for modern varieties, L1, L2 and L3 are the three groups identified for landraces. Note that some landraces were assigned as Lmixed group because the results from both analyses were not congruent.**
